# Challenges and approaches for production of a healthy and functional mayonnaise sauce

**DOI:** 10.1002/fsn3.1132

**Published:** 2019-07-18

**Authors:** Mina Mirzanajafi-Zanjani, Mohammad Yousefi, Ali Ehsani

**Affiliations:** ^1^ Student Research Committee, Department of Food Science and Technology Tabriz University of Medical Sciences Tabriz Iran; ^2^ Department of Food Science and Technology, Food and Drug Safety Research Center Tabriz University of Medical Sciences Tabriz Iran

**Keywords:** fat replacer, functional, healthy, low cholesterol, mayonnaise, natural preservative, probiotic

## Abstract

Mayonnaise is a semisolid oil‐in‐water (O/W) emulsion which is made through the careful blending of oil, vinegar, egg yolk, and spices (especially mustard). In addition, mayonnaise traditionally contains 70%–80% oil, and egg yolk is a key ingredient contributing to its stability. Despite concerns about high cholesterol level in egg yolk, it is yet the most widely utilized emulsifying agent owing to its high emulsifying capacity. Today, the public knowledge about diet and health has been incremented, compelling the people to consume foodstuffs containing functional features. Thus, consumers, aware of the considerable influence of the diet on their health, demand nutritious and healthier food. Mayonnaise is usually cited by health‐related issues due to its high cholesterol and fat content. Many researchers have tried to replace fat, as well as egg yolk completely or partially; however, low‐fat mayonnaises require extra ingredients to keep the stability. In other words, each ingredient plays a specific role in textural and oxidative stability, and using alternative emulsifiers and fat replacers may affect the sensorial, textural, and antioxidant features of mayonnaise. Furthermore, mayonnaise, like other high‐fat foodstuffs, is vulnerable to auto‐oxidation. In addition to using fat replacers, mayonnaise is accompanied with bioactive ingredients to produce a healthy system. Therefore in this review, we gathered a quick summary of the ideas, including lowering the cholesterol and fat and using natural antioxidants, prebiotics, and probiotics in order to produce a healthy and functional mayonnaise sauce.

## 1 INTRODUCTION

The development of new food products seems to be increasingly challenging, because it has to comply with the consumers’ satisfaction, especially for relish health foodstuffs. In this concern, functional foods that have health benefits in addition to nutritional contents and particularly foods with reduced fat are of great importance (Bigdelian & Razavi, 2014; Miele, Di Monaco, Cavella, & Masi, 2010). From the marketing point of view, it is vital to know about the importance of the health claim of functional foods.

Food fulfills three principal functions: the first one is nutrition, followed by reducing lifestyle‐related illnesses. These two functions were primarily described in 1984 in the deployment and systematic analysis of functional food research project funded by the Ministry of Education, Culture, Sports, Science, and Technology, Japan. The third function, defined in terms of foodstuffs with health claims (FHCs), was first labeled in Japan, which established regulations for expanding FHCs. Nowadays, the consumption of functional foods has spread throughout the world and been encouraged by the increasing dietary interest of consumers. Consumers demand to purchase functional foods, in which they recognize the health‐promoting features, not found in conventional foods.

Over the past decades, the number of studies, pertaining the low‐fat edible products, has increased with considerable regard to diseases such as obesity, cardiovascular diseases, and cancer (Chang et al., 2017). Fat, as a substantial constituent of foods, has long been noted as the main source of energy and the satiety. Gradually, other benefits of edible products as fat‐soluble vitamin carriers and major sources of essential fatty acids were likewise recognized (Emadzadeh & Ghorani, 2015). However, on account of lifestyle changes and the lack of any balance between the intake and expenditure of energy, obesity has incremented globally (Aganovic, Bindrich, & Heinz, 2018). Considering the over‐consumption of fat as a deciding factor in obesity, the production of low‐fat foodstuffs has stimulated many research interests (Ma & Boye, 2013). In this regard, no‐fat and low‐fat sausages, cream, yoghurt, and mayonnaise have been developed (Sun et al., 2018). Regarding this, mayonnaise manufacturers now tend to produce low‐fat mayonnaise, because oil is commonly the most expensive ingredient of mayonnaise (Depree & Savage, 2001).

Although replacing the fat is the crucial part of producing healthy mayonnaise, in these days, consumers demand not only a healthy food, but also a functional product, a product that can respond to medical needs of people beyond their nutritional requirements. To this end, several beneficial ingredients, such as probiotics, prebiotics, antioxidants, and phytosterols, have been added to mayonnaise. Thus, this paper mainly aimed to describe the properties of functional mayonnaise, as well as explaining the role of its different ingredients.

## 2 MAYONNAISE INGREDIENTS AND FEATURES

Mayonnaise is an oil‐in‐water (O/W) emulsion and is widely consumed as a traditional seasoning due to its creamy mouthfeel and special flavor. The conventional mayonnaise contains 65%–80% fat, which contributes to its texture, appearance, flavor, and shelf life (Sun et al., 2018; Worrasinchai, Suphantharika, Pinjai, & Jamnong, 2006). Mayonnaise is presumed to have originated from Port Mahon, France, in 1756. It was produced for celebrating the conquering the Port Mahon by forces under the command of Louis Francois Armand de Vignerot du Plessis, duc de Richelieu (1696–1788), a marshal of France, and it was called Maho´nnaise. The word was later changed to mayonnaise, probably because of the old French words for egg yolk and to stir, moyen and manier (Morley, 2016).

**Figure 1:**
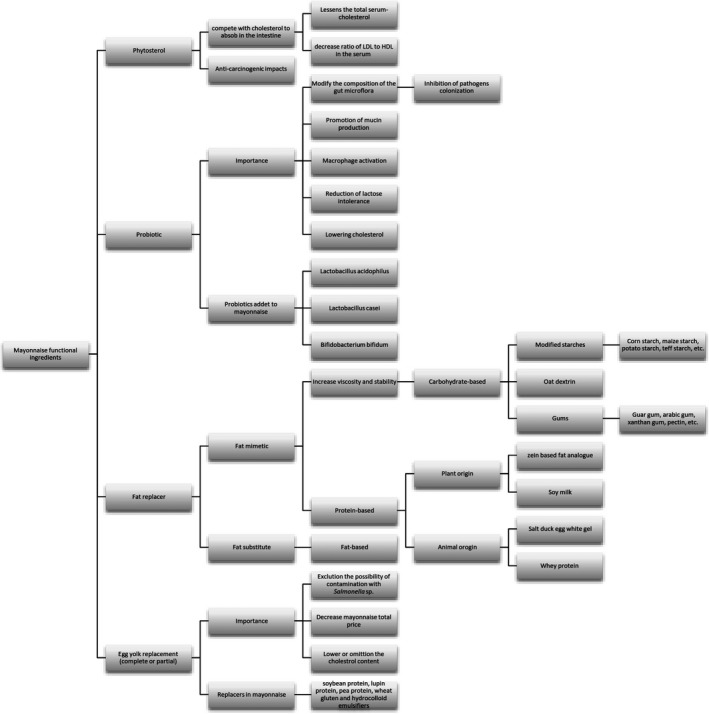
Main constituents of mayonnaise and their roles

Mayonnaise was produced commercially in the early 1900s for the first time and then became popular in America from 1917 to 1927 (Harrison & Cunningham, 1985). Later in Japan, the mayonnaise price was incremented by 21% from 1987 to 1990 (Le, 1992). This emulsion includes an aqueous solution as a constant phase and oil as a dispersed phase (Aganovic et al., 2018). It is produced using vegetable oil, emulsifier (egg lecithin), acidic components (acetic acid, citric acid, and maleic acid), flavoring agents (sweetener, salt, mustard, or garlic), texture enhancers, stabilizers, and an inhibitor for unwanted crystals (Yildirim, Sumnu, & Sahin, 2016). Some features of mayonnaise ingredients are illustrated in Figure 1.

### 2.1 The role of oil fraction

The mayonnaise emulsion is formed by leisurely blending of the oil with a premix, including vinegar, mustard, and egg yolk, because blending the aqueous phase and oil at once would result in the creation of a water‐in‐oil emulsion (Liu, Xu, & Guo, 2007). Traditional production of the emulsion often includes batch mixers, meaning that the oil is gradually added to an aqueous phase under extreme mixing, though production by the use of a high speed mixer and the batch process is fairly inefficient (Depree & Savage, 2001). Continuous procedures are available for making mayonnaise as well. In these methods, there are pumps, which blend three phases of egg, oil, and water in a mixer, following continuous homogenization. Furthermore, a batch‐continuous method exists, which is a mixture of both procedures, where coarse‐form emulsion is made batch‐wise, following continuous steps to decrease the fat droplets (Aganovic et al., 2018).

Mayonnaise is a microbe‐stable foodstuff due to its high fat content and acidic conditions and may be kept at room temperature; nevertheless, the loss of quality always exists owing to the auto‐oxidation of unsaturated fatty acids (Aganovic et al., 2018). Fat, as one of the major ingredients, positively affects the rheological attributes and sensory characteristics of the final produced mayonnaise. It also contributes to the flavor, texture, creaminess, palatability, appearance, and shelf life of mayonnaise (Mun et al., 2009). Moreover, one of the most important features of mayonnaise, basically induced by fat, is the mouthfeel property. Generally, the mouthfeel for fat in a lipid‐based product is a rheological phenomenon. The sensation of fattiness is a complex phenomenon, involving flowability and viscosity properties of a food product. Ma et al. studied the functionality of fat replacers in foods and discovered that particles smaller than 3 µm in diameter could not be distinguished by the human tongue (Ma, Cai, Wang, & Sun, 2006).

### 2.2 The role of salt and vinegar

The most important role of vinegar is pH adjustment. The mayonnaise pH has a profound impact on the emulsion structure. The stability and viscoelasticity of mayonnaise would be at its highest point when the pH value reaches the isoelectric point of egg yolk proteins, to such a degree that the proteins’ surface charge is lessened. The flocculation of proteins would never happen if the proteins are highly charged (Depree & Savage, 2001).

Mayonnaise oil droplets are positively charged owing to the composition of proteins in the interface, as well as the medium pH (<4.2 for mayonnaise). It has been proved that droplets containing negative charge tend to adsorb metal ions with positive charge, which can favor the development of lipid oxidation (Aleman et al., 2015). In addition, a low pH value (pH = 4) breaks the ion bridges present between phosvitin and iron. Moreover, ferric ions are insoluble and soluble in neutral and low pH values, respectively. Therefore, decreasing the pH value may also render the incremented solubilization of iron ions in the mayonnaise (Jacobsen, Timm, & Meyer, 2001), which is important from the oxidation point of view.

From the microbiological viewpoint, Xiong, Xie, and Edmondson (2000) suggested that at least 60 ml vinegar per fresh whole egg, 40 ml per fresh egg white, or 20 ml per fresh egg yolk (6% w/v acetic acid) is required to produce *Salmonella‐*free mayonnaise in the kitchen.

Concerning the salt, its addition can enhance the mayonnaise characteristics for three main reasons. First, salt aids in dispersing the egg yolk granules and increasing the availability of more surface‐active materials. Second, salt neutralizes protein charges, so the proteins can easily be adsorbed to the surface of the oil droplets. Third, it provides the proximity of oil droplets to each other, thereby interacting more strongly. However, excessive salt may trigger the aggregation of egg yolk proteins in the aqueous phase owing to the salting‐out effect (Depree & Savage, 2001).

### 2.3 The role of egg and its substitution challenges

Egg is well‐known for its gelling, whipping, and emulsification features. It plays a significant role in the preparation of foods. The three most known usages for eggs are as follows: liquid egg, which solidify or coagulate when heated (in order to solidify and produce cakes and so on); aeration or whipping which generates lighter and airier products (e.g., merengue); and the emulsifying phospholipids existed in egg yolk and lipoproteins, which would make sauces and salad dressings (Abu‐Salem & Abou‐Arab, 2008).

In the mayonnaise recipe, egg yolk is the most vital part for the stability of the emulation (Nikzade, Tehrani, & Saadatmand‐Tarzjan, 2012). Egg yolk has wonderful quality for forming the mayonnaise emulsion and for preventing the flocculation to form an appropriate texture (Depree & Savage, 2001). Furthermore, the high emulsifying potential of egg yolk is related to the LDL (low‐density lipoprotein), HDL (high‐density lipoprotein), phospholipids, and nonbonded proteins (phosvitin and livetin) (Laca, Sáenz, Paredes, & Díaz, 2010; Moros, Franco, & Gallegos, 2002).

Although egg possesses brilliant emulsifying property, the constraints are the possibility of contamination with *Salmonella* sp., and price, as well as high cholesterol content of egg yolk (Smittle, 2000). For these concerns, scientists have researched into the role of animal proteins to replace the egg yolk. Therefore, emulsification properties of animal proteins such as casein, whey protein, and meat protein have been widely investigated by a number of researchers (Nikzade et al., 2012). In addition to animal protein, plant ones have also been considered. The utilization of plant proteins is needed to support the production of protein‐rich foods which can find itself properly in the human diet. Hence, the use of plant proteins (e.g., lupin protein, soybean protein, and pea protein) instead of egg yolk to stabilize the oil‐in‐water emulsion is the most popular method for preparing mayonnaise‐like emulsions (Diftis, Biliaderis, & Kiosseoglou, 2005). For example, starch paste has been used to replace egg yolk (Dolz, Hernández, & Delegido, 2006; Mancini, Montanari, Peressini, & Fantozzi, 2002). However, the usage of starch paste extends the duration and price of processing along with an unfavorable possible effect on the texture and flavor of mayonnaise. The other approach is the use of egg yolk containing low cholesterol as the emulsifying agent in mayonnaise (Laca et al., 2010).

## 3 CONDITIONS USED TO PRODUCE HEALTHY AND FUNCTIONAL MAYONNAISE

**Figure 2:**
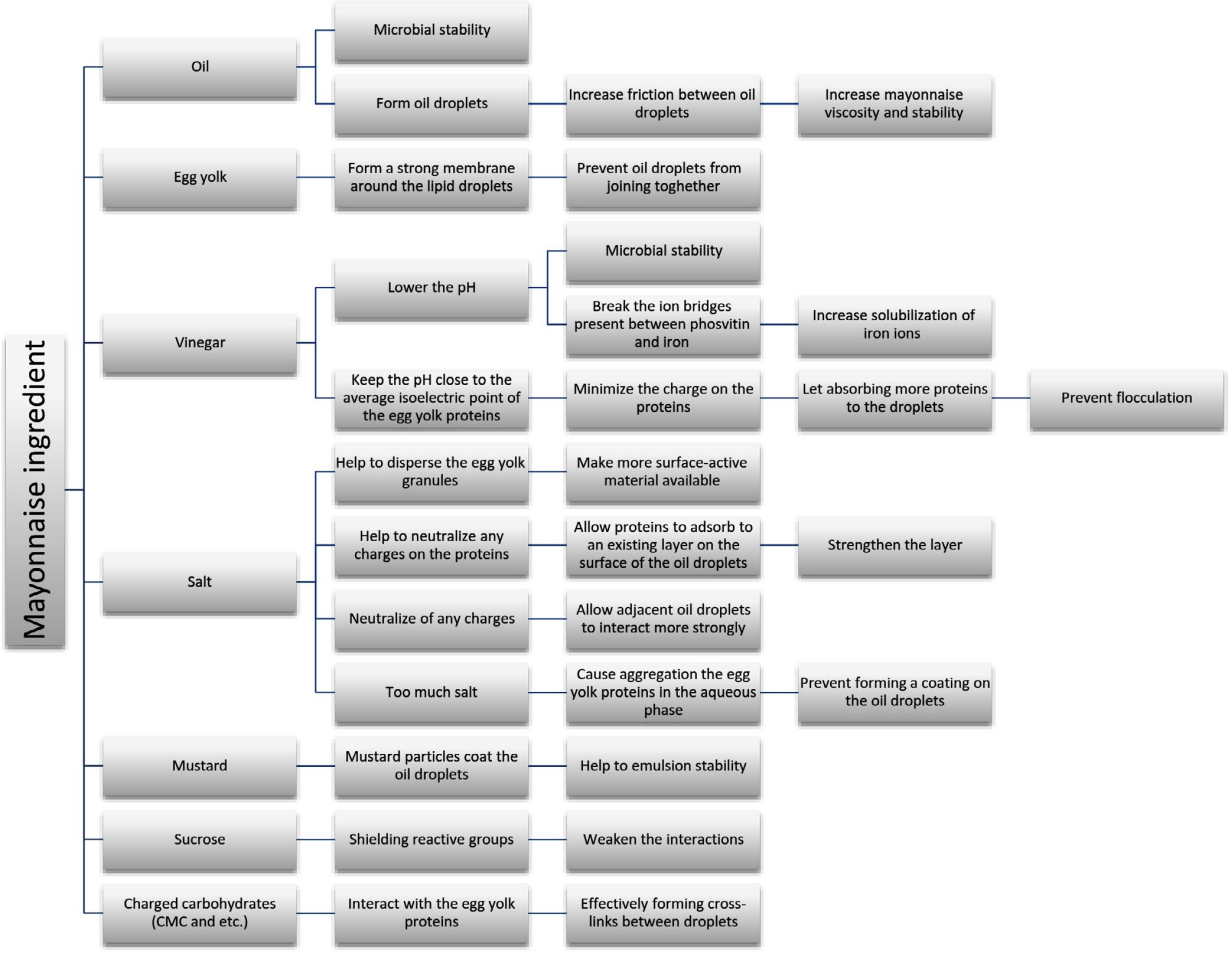
Functional ingredients added to mayonnaise and their importance

The investigation of researchers demonstrates that two main approaches are considered in producing a healthy and functional mayonnaise: using fat replacers, or/and adding some functional ingredients to mayonnaise, which usually are composed of prebiotic, antioxidant, or phytosterol (Figure 2). The application of these ingredients in mayonnaise is discussed in the following parts.

### 3.1 Replacement for fat/oil

The American Heart Association has suggested limiting the fat usage to lower than 30% of the whole consumed calories (Amin, Elbeltagy, Mustafa, & Khalil, 2014). In addition, the substitution of a part of fat without decreasing the taste is a key factor in producing low‐fat foodstuffs (Santipanichwong & Suphantharika, 2007). Moreover, the sensory and physiochemical properties of mayonnaise are significantly affected by the elimination of fat; hence, the attention to fat replacers is increasing (Chung, Degner, & McClements, 2014). From a physical point of view, decreasing the dispersed phase and increasing the water content are necessary to create a low‐fat emulsion (Izidoro, Scheer, Sierakowski, & Haminiuk, 2015).

Unfortunately, decreasing the oil proportion in mayonnaise decreases the oil droplets density, thereby weakening the stability and interactions between droplets and emulsion. In addition, stability of low‐fat emulsions can get better through decreasing the droplet size, which also provides a “creamier” appearance (Depree & Savage, 2001). In another word, reducing the fat level would result in the increment of the water content and aqueous phase, as well as inducing the decrease in the firmness and viscosity of emulsion. Furthermore, fat substitutes are used to produce mayonnaise with a texture near to those of traditional ones (Chang et al., 2017). Viscosity, in a low‐fat mayonnaise, is incremented by additives, especially hydrocolloids, which would result in the increase of density and stability of the emulsion by reducing the coalescence (Karas, Skvarča, & Žlender, 2002). To replace fats, substances mostly should hold the following attributes: empty of osmotic and diarrhea effects, toxicologically safe, functionality analogues to fat, mouthfeel‐like fat, and a similar price to fat (Cheung, Gomes, Ramsden, & Roberts, 2002). Different roles of fat replacers in mayonnaise are summarized in Table 1.

**Table 1:** Different roles of fat replacers in mayonnaise

Fat replacer	Optimum used percentage of fat replacer	Results	References
Durian (*Durio zibethinus* L.) seed gum	4%	Fairly stable emulsion, good texture, and not too large fat globule size	Cornelia, Siratantri, and Prawita (2015)
Wheat gluten	1.0 wt.%	Very similar droplet size distribution, similar sensory property, and texture such as creaminess, smoothness, and sliminess to control mayonnaise	Liu et al. (2017)
Inulin with short, medium, and long chained and modified starch	Inulin with short, medium, and long chained (0%–10%) and modified starch (1.5%)	Increased gel strength	Alimi et al. (2013)
Soy soluble polysaccharide, gum Arabic	40%	Very similar rheological features especially for OSA‐S stabilized emulsions	Chivero, Gohtani, Yoshii, and Nakamura (2016)
Micronized desalted egg white gel	30%	Lower calorie and higher storage stability	Wang et al. (2015)
Microparticulated whey protein (MWP) and high‐methoxyl pectin	60%	Weaker gels except the 20% fat substituted sample which displayed high storage stability	Sun et al. (2018)
Pectin sol (PS) and egg white protein microparticle (EWPM)	In weight ratio 2:4 of PS and EWPM	Yellow value of light mayonnaise increased, thermal resistance decreased	Chang et al. (2017)
Oat dextrin	27.9%	Fat granules became uniform, small, and symmetrical	Shen et al. (2011)
Whey protein isolate and low‐methoxyl pectin	50%	Similar texture values as the full‐fat samples	Liu et al. (2007)
Sodium octenyl succinate starch	50%	Without effect on the mean droplet size and phase separation. L* value of treated samples was significantly higher than those of full‐fat mayonnaises.	Thaiudom and Khantarat (2011)
Micronized konjac gel	4.0 wt.%	Good storage stability	Li et al. (2014)
Soy milk, xanthan gum, guar gum, and mono‐ and diglycerides	6.7% mono‐ and diglycerides, 36.7% guar gum, and 56.7% xanthan gum	An increase of xanthan gum followed by guar gum caused greater values for the stability, heat stability, consistency, viscosity, firmness, adhesiveness, adhesive force, and overall acceptance	Nikzade et al. (2012)
β‐Glucan	50%	Samples showed similar firmness and adhesiveness values as control mayonnaise	Worrasinchai et al. (2006)
*Opuntia robusta* mucilage and whey protein	62.50% mucilage and 10.71% whey protein	Oxalate calcium crystals were present in the *Opuntia robusta,* and mucilage with druses morphology was not observed due to the product acidic pH; so it is suitable to develop a functional low‐fat mayonnaise.	Bernardino‐Nicanor et al. (2015)
Octenyl succinic anhydride (OSA), modified corn, and white sorghum starch	75%	Overall, acceptability increased compared to control samples with the same textural quality	Ali et al. (2015)
Sodium alginate, xanthan gum, guar gum, and carboxymethyl cellulose (CMC)	50%	Providing a high viscous emulsion	Thomareisa and Chatziantoniou (2011)
Zein‐based fat analogue	40%	Good appearance, stability, and total calorific value, as well as the rheological, microstructural, and sensorial results	Gu et al. (2016)
Xanthan gum, citrus fiber, and guar gum (GG)	50%	No rheological and sensorial differences between treated and control mayonnaises	Su, Lien, Lee, and Ho (2010)

### 3.2 Fat mimetic

Fat replacers are categorized into three groups: carbohydrate‐, protein‐, and fat‐based replacers. Among the replacers, fat‐based replacers are known as fat substitutes and carbohydrate‐ and protein‐based ones are generally called fat mimetics, which cannot fully replace the fat in the foods, but offer similar mouthfeel and texture to fats as well as great amount of dietary polysaccharides and proteins (Sun et al., 2018). Konjac gel (Li, Wang, Jin, Zhou, & Li, 2014), salt duck egg white gel (Wang, Zheng, Li, Li, & Chen, 2015), 4aGTase‐modified rice starch (Mun et al., 2009), oat dextrin (Shen, Luo, & Dong, 2011), modified starch (Ali, Waqar, Ali, Mehboob, & Hasnain, 2015), inulin (Alimi, Mizani, Naderi, & Shokoohi, 2013), pectin (Chang et al., 2017), and some thickeners have been investigated to replace fat in mayonnaise. In the meantime, there is an interesting point about pectin, because it has the potential to inhibit the digestion of lipid along with increasing the stability and reducing the creaming of oil droplets (Sun et al., 2018).

Moreover, whey protein is commonly utilized in the production of fat mimetics because of its ability in the coagulation of a gel under distinct temperature and pH circumstances. Microparticulated whey protein (MWP) covers taste buds analogues to lipids. This coating manner helps flavors to gradually reach the receptors and helps to mask some astringent and bitter flavors in low‐fat products (Chung et al., 2014).

Sun et al. (2018) used MWP‐pectin complex to produce low‐fat mayonnaise; this partially suppressed the viscoelasticity reduction (substitution of fat up to 40% was acceptable). The decrease of fat up to 20%, 40%, 60%, and 80% reduced the caloric values up to 17.6%, 35.4%, 53.1%, and 71.4%, respectively. The full‐fat mimetic product showed the lowest value of calorie (%0 oil, 83.52 kcal/100 g) with nearly 90.0% decrease at the caloric value. Sensory evaluations, likewise, demonstrated that MWP‐pectin complexes have the merit to replace 40% of lipid in order to make a low‐fat mayonnaise with the color, texture, appearance, odor, and taste similar to full‐fat mayonnaise, but containing lower calorie.

Teff, an indigenous tropical cereal crop of Ethiopia, is a good source of starch. Teff starch contains 2–6 mm granules, with a little dissimilar properties in comparison with other tropical starches, and has been suggested as a good fat mimetic. Teklehaimanot, Duodu, and Emmambux (2013) investigated the impact of maize and teff starch pastes amended by stearic acid on the microstructure and rheological characteristics of low‐fat mayonnaise. They reported that both of modified and unmodified maize and teff starches could produce low‐fat mayonnaises at 50% replacement for oil. By replacing the oil up to 80%, only the modified products had physical properties similar to full‐fat products. This replacement could meaningfully decrease the calorific value as much as 76.44%.

In another study, Mun et al. (2009) showed that the mayonnaise lipid can be moderately substituted (50%) by the 4‐glucanotransferase (4GTase)‐treated starch blended with xanthan gum. The results indicated that 4GTase could modify the polymers of starch and produce thermoreversible gels through decreasing the amylose structure as well as modifying the amylopectin side chains. In fact, this enzyme attacks the ‐1,4‐ glucosidic bond and conveys a fragment of glucan donor molecule to a glucan acceptor through creating a new ‐1,4‐glucosidic linkage, leading to the formation of exclusive amylopectin clusters from starch molecules. This study indicated that the usage of 0.1 wt% of xanthan gum and 5.6 wt% of 4aGTase‐treated starch made a low‐fat mayonnaise with analogous appearances and rheological attributes to the full‐fat mayonnaise. The findings illustrated that when the amount of 4GTase‐treated starch and xanthan gum added to the reduced‐fat mayonnaise is under control, the final product would be used as a good fat replacer of mayonnaise.

### 3.3 Antioxidants

Oxidation reactions are considered as interfacial phenomena, which are influenced by a number of different parameters such as physicochemical properties of water and oil phases, chemical composition, the kind of surfactants, and the oil phase surface area (Kishk & Elsheshetawy, 2013). In addition, interfacial oxidation is a critical issue about emulsified foods such as mayonnaise, because it affects the shelf life of the food (Calligaris, Manzocco, & Nicoli, 2007). Moreover, mayonnaise is at risk of lipid oxidation when kept at 4°C (Raikos, Neacsu, Morrice, & Duthie, 2015). Mayonnaise oxidative stability is largely affected by its ingredients and especially the kind of oil. For example, the production of mayonnaise with n‐3 polyunsaturated fatty acids (PUFAs) increases the possibility of oxidation. Although PUFAs have nutritional and health benefits, their oxidation leads to the development of reactive aldehydes, free radicals, off‐flavor, and the decrease in the shelf life of mayonnaise (Aleman et al., 2015).

Normally, synthetic antioxidants of TBHQ, BHA, and BHT are utilized to suppress the rancidity of fats. Although the antioxidant strength of these synthetic antioxidants is more than that of natural antioxidants in many cases, the toxicity of these antioxidants and consumer demands for natural products have turned the attention toward the use of natural antioxidants (Kishk & Elsheshetawy, 2013). Natural antioxidants originate from several marine algae and plants, many of which display a high potential to enhance the stability of foodstuffs against oxidation. Moreover, these antioxidant substances have a wide spectrum of health‐promoting advantages (Hermund et al., 2015).

In a study, Jacobsen, Hartvigsen, et al. (2001) investigated the antioxidant impacts of EDTA gallic acid, and extra Panodan DATEM TR as emulsifiers in mayonnaise incorporated with 16% fish oil. EDTA decreased the production of lipid hydroperoxides, free radicals, and rancid and fishy off‐flavors. These results were attributed to the chelation of iron and free metal ions by EDTA from egg yolk. Gallic acid decreased concentrations of lipid hydroperoxides and free radicals, but increased slightly the oxidative off‐flavor. Finally, the addition of extra emulsifier decreased only the level of lipid hydroperoxide, but did not affect the concentration of free radicals or the off‐flavor in mayonnaise.

Honold, Jacobsen, Jónsdóttir, Kristinsson, and Hermund (2008) considered the potential of seaweed‐based food antioxidants to delay the oxidation of lipid in the mayonnaise enriched with fish oil. In this study, acetone, ethanol, and water were used to extract the phenolic contents of *F. vesiculosus*. Ethanol and acetone successfully extracted high concentrations of carotenoids and phenolic compounds. In addition, water was found to not only extract some phenolic substances, but also to extract higher concentrations of chlorophyll derivates and metals. Results showed that ethanol and acetone extracts had the highest antioxidant capacities.

The potential to chelate trace metals is also an important parameter of antioxidant activities, specifically in the mayonnaise (Honold et al., 2008). The trace metals like copper and iron may interact with unsaturated fats to produce radicals (alkyl radicals) or cause peroxides degradation to alkoxyl radicals. A large value of iron in mayonnaise is originated from the egg yolk. In egg yolks, the chief proportion of iron has been bonded to the phosvitin. However, by the incorporation of egg yolk to mayonnaise, the low pH (pH = 4) breaks the ion bridges between phosvitin and iron, thereby enabling the participation of iron in lipid oxidation (Hermund et al., 2015). The synthetic chelating agents like EDTA have been observed to be the highest effective inhibitors against metal‐catalyzed oxidation in mayonnaise (Jacobsen, Hartvigsen, et al., 2001).

In addition, Sørensen, Nielsen, Hyldig, and Jacobsen (2010) acclaimed that the type of emulsifier is another substantial factor in mayonnaise oxidation. They investigated the stability of mayonnaise enriched with fish oil (40% oil) against oxidation as well as the influence of egg yolk and milk protein‐based emulsifier. Furthermore, the impacts of different levels of fish oil (4%, 10%, and 14%) and storing temperatures (2 and 20°C) were explored. The findings demonstrated that the lipid oxidation was incremented by increasing the fish oil level and storage temperature. Surprisingly, the substitution of egg yolk with a milk protein‐based emulsifier (a less iron‐containing emulsifier) could not significantly increase the oxidative stability of samples. Moreover, the peroxide value obtained for milk protein‐based emulsifier was nearly 100‐fold higher than that obtained for egg yolk. In this regard, the weaker oxidative stability of samples containing milk protein‐based emulsifier was attributed to the primary quality of the emulsifier.

Concerning the importance of emulsifier type, Jacobsen, Timm, et al. (2001) suggested the following mechanism for the oxidation of lipids, in which lowering the pH value to near 4 in mayonnaises enriched with fish oil breaks the iron bridges between low‐density lipoproteins (LDLs), phosvitin, and lipovitellins, and consequently releases iron from egg yolk. Subsequently, this iron may catalyze the oxidation of lipids in the mayonnaise. Based on these findings, it can be hypothesized that the substitution of egg yolk with the emulsifiers containing a decreased iron content might heighten the oxidative stability of mayonnaise.

One of the most widely used approaches to deal with mayonnaise oxidation is adding vegetables, containing high antioxidant capacity. For example, Raikos et al. (2015) supplemented mayonnaise with some vegetables (5% w/w) and investigated the impact of storing time at 4°C on the stability of dispersed phase against oxidation. Results inferred from both TBARS and Rancimat indicated that the vegetable type utilized for the reformulation of mayonnaise is substantial in the inhibition of oxidation, and followed the order beetroot > carrot ≈ onion regarding the antioxidant capacity. *Fucus vesiculosus* (Hermund et al., 2015), lipophilized caffeine (Aleman et al., 2015), ginger powder (Kishk & Elsheshetawy, 2013), eugenol‐lean clove extract (Chatterjee & Bhattacharjee, 2015), and microwaving‐processed beetroot (Raikos, McDonagh, Ranawana, & Duthie, 2016) are other examples.

### 3.4 Prebiotics

The definition of prebiotic, according to Gibson and Roberfroid, is “selectively fermented ingredient that allows specific changes; both in the composition and/or activity of the gastrointestinal microbiota that confers benefits upon host well‐being and health” (Gibson, Probert, Loo, Rastall, & Roberfroid, 2004; Roberfroid, 2007). Conferring to this definition, prebiotics must fulfill the following criteria in in vitro and in vivo tests: (a) nondigestibility (resistance to enzymatic digestion, gastric acid, low pH, and intestinal absorption); (b) fermentable by intestinal microbiota; and (c) selective growth and activity of certain intestinal microorganisms (de Vrese & Schrezenmeir, 2008). Fructans such as inulin and fructooligosaccharides (FOS), cyclodextrins (CDs), and galactooligosaccharides (GOS) are some of the most known prebiotics (Choque Delgado & Tamashiro, 2018). The benefits of the consumption of prebiotics are as follows: producing polyamines and short chain fatty acids (Ooi, Correa, & Pak, 2019), enhancing the motility and gastrointestinal performance, decreasing cholesterol, stimulating the immune system (Roberfroid, 2008), reducing the lipid plasma levels, and improving energy homeostasis (Glenny, Bulik‐Sullivan, Tang, Bulik, & Carroll, 2017). In addition, various experiments have proved that prebiotics can help in reducing the severity of particular diseases such as hepatic encephalopathy (Floch, 2018), obesity (Kao, Burnet, & Lennox, 2018), diabetes, inflammatory bowel disease (IBS), neural disorders, and other infectious diseases (Maguire & Maguire, 2019). Prebiotics also play an imperative role in modulating the expression of genes and have a high impact on human metabolism to control diabetes mellitus type two.

According to Global Market Insights, INC, the global market of prebiotics is expected to surpass 8.5 billion US dollars by 2024 (Fonteles & Rodrigues, 2018). Statistics show that more than five hundred new products enriched with prebiotics have been offered to the market during the past few years (Silveira et al., 2015).

Inulin is one of the most recently used prebiotics in the mayonnaise formulation. Along with the prebiotic property, inulin forms insoluble crystals in food systems when contacting with water, causing a unique gel structure and making a spreadable texture (Franck, 2002). In addition, inulin particles function in the same way to oil droplets in O/W emulsions and can be used as the fat replacer (Alimi et al., 2013; Bot, Erle, Vreeker, & Agterof, 2004). Aganovic et al. utilized the combination of inulin (12 wt%) and high‐pressure homogenization (HPH) technology to develop a functional mayonnaise sauce. The gelling behavior of inulin while using high homogenization forces formed emulsions with better rheological properties (viscoelasticity and viscosity), compared to rotor–stator. This effect could result in the long‐term stability, reducing oil creaming of the emulsion (Aganovic et al., 2018).

In another research, Liutkevičius et al. evaluated the effect of chitosan as a prebiotic dietary fiber on the quality and microbiological characteristics of mayonnaise and observed that chitosan meaningfully incremented the values of samples acidity. Adding chitosan did not have a significant impact on the total plate count of bacteria and growth of fungi in mayonnaise as well as a slight change in texture properties. However, adverse effects were seen pertaining the sensory properties of the samples which caused a reduction in the taste and odor (Liutkevičius et al., 2014).

### 3.5 Probiotics and the effects of encapsulation by prebiotics

Food and Agricultural Organization of the World Health Organization has defined probiotics as “live microorganisms which when administered in adequate amounts confer a health benefit on the host” (Hill et al., 2014). Like prebiotics, probiotics can amend the arrangement of the gut microflora and affect both intestinal and body functions (Roberfroid, 2010). The consumption of probiotics affects various aspects of immune system such as increasing mucin production, inhibiting pathogens colonization, decreasing gut permeability, and activating macrophages and natural killer cells. Concerning the adaptive immune system, the observed effects include the increase in the antibodies (IgA, IgM, and IgG) production, and an arrangement in the branches of the immune system through the production of cytokines (Anadón, martinez‐larrañaga, ArÉS, & MartÍNez, 2016).

Probiotic bacteria such as lactobacillus and bifidobacteria have therapeutic performances by lowering cholesterol, preventing cancer, alleviating constipation, and reducing lactose intolerance (Guerin, Vuillemard, & Subirade, 2003). Yet, to make these functions, probiotics should be stable while passing the gastrointestinal tract, along with colonizing in the intestine (Brinques & Ayub, 2011). For applying these advantages, probiotics should be added at the concentration of 10^6^ CFU/g to the products (Chan & Zhang, 2002). Mayonnaise sauce can be a suitable carrier for probiotics owing to its high water activity (Fahimdanesh et al., 2012). Foods containing high buffering capacity increment the pH of the gastric tract and result in improving the stability of probiotics, thereby making mayonnaise a good matrix for probiotics (Shen et al., 2011).

Furthermore, probiotics are not usually used in direct form in foodstuffs due to the sensorial and stability issues (de Vos, Faas, Spasojevic, & Sikkema, 2010); therefore, many researchers are trying to find a way to increase the survival of probiotic cells. To protect probiotics against adverse environmental, processing, and intestinal conditions, these cells have to be protected with a physical barrier (Kailasapathy, 2009; Schell & Beermann, 2014). Microencapsulation is considered a useful method to protect probiotics (Burgain, Gaiani, Linder, & Scher, 2011).

To this end, many biopolymers are utilized to coat the probiotics. For instance, alginate as one of the mostly used biopolymers for microencapsulation has been observed to increase the survival of probiotic cells from 80% to 95% (Mandal, Puniya, & Singh, 2006). Alginate is a cheap, biocompatible, and nontoxic substance to the body (Chávarri et al., 2010; Kasra‐Kermanshahi, Fooladi, & Peymanfar, 2010). This biopolymer is an anionic polysaccharide, constituted of glucuronic acid and D‐mannuronic acid residues linked by 1–4 glycosidic linkages (Annan, Borza, & Hansen, 2008). Incorporating both prebiotics and prebiotic coating materials may improve the protection of probiotics in foodstuffs and even in the gastrointestinal tract due to symbiosis, called “symbiotic” product (Chen, Chen, Liu, Lin, & Chiu, 2005; Nazzaro, Fratianni, Coppola, Sada, & Orlando, 2009).

Khalil and Mansour (1998) added *Bifidobacterium bifidum* and *Bifidobacterium infantis* to mayonnaise as free and alginate encapsulated cells. The viability of free cells was totally destroyed after 2 weeks; nevertheless, encapsulated *B. bifidum* survived up to 12 weeks and *B. infantis* for 8 weeks (Khalil & Mansour, 1998).

Bigdelian and Razavi (2014) evaluated the survival of two strains of *L. acidophilus* and *L. casei* in three types of symbiotic mayonnaise sauces, with free, encapsulated bacteria with calcium alginate (in 4% concentration) and encapsulated with calcium alginate and resistant starch during 91 days. According to the results, microencapsulation could help the survival of probiotic cells, and adding Hi‐maize starch to calcium alginate improved preserving the chemical qualities of symbiotic mayonnaise sauce, since it does enhance the survival of the probiotic bacteria. Viable cells of *L. acidophilus* mixture with mayonnaise sauce showed 2.659 log decreases for the free state, while the encapsulated state with alginate (4% concentration) decrease of 1.48 logs, the encapsulated bacteria (*L. acidophilus*) with Hi‐maize starch, and alginate mixtures decreased about 1.1497 logs after 91 days.

Mohammadi et al. (2012) considered the survival of alginate‐prebiotic resistant starch‐microencapsulated *Lactobacillus acidophilus* in mayonnaise sauce. This study indicated that the resistant starch could give a higher viability of *L. acidophilus* (10^5^ to 10^6^/g) in acidic condition, and the loss of *L. acidophilus* cells showed significant differences (*p* < .05) between the free and encapsulated states in mayonnaise sauce about 2 logs at the end of 30 days storage (Mohammadi et al., 2012).

Fahimdanesh et al. (2012) added *Lactobacillus casei* and *Bifidobacterium bifidum* to mayonnaise in the forms of free or encapsulated cells with resistant starch and assessed their viabilities. The results indicated that microencapsulation with resistant starch improved the viability of *L. casei* and *B. bifidum* in comparison with free cells during 30 days storage. Furthermore, microencapsulation with resistant starch could preserve the sensory quality of the mayonnaise sauce better than the samples containing free probiotics (Fahimdanesh et al., 2012).

### 3.6 Natural preservatives used in mayonnaise

Mayonnaise sauce is a relatively microbial safe product owing to its high fat content and presence of acidic ingredients which reduce the pH of product to a lower value of 4.8 (Depree & Savage, 2001; Karas et al., 2002). Most pathogenic bacteria such as *Escherichia coli*, *L. monocytogenes*, *Salmonella*, *Yersinia enterocolitica*, and *Staphylococcus aureus* are destroyed when inoculating into mayonnaise. However, spoilage microorganisms such as *lactobacilli* might grow in mayonnaise and affect its shelf life and safety (Fialová, Chumchalová, Miková, & Hrůšová, 2008). In addition, certain organisms such as *E. coli* can be broken out by mayonnaise. Furthermore, the colonization of microbes in mayonnaise differs based on the type of acid used, temperature, pH, and storage time (Yolmeh, Habibi Najafi, Farhoosh, & Salehi, 2014).

In these days, the usage of natural preservatives instead of synthetic ones is promising, because synthetic preservatives are suspended to be nonsafe and possibly harmful. To control the growth of microorganisms, organic acids such as sorbic acid and benzoic acid and/or a mixture of them have usually been advised as the most applicable preservatives in mayonnaise. The maximum allowed concentrations of sorbic acid and benzoic acid, in this regard, are 1 g/kg and 2 g/kg of mayonnaise, respectively. However, these preservatives are not capable of controlling the growth of *lactobacilli* (Yolmeh et al., 2014). Along with organic acids, some naturally occurring preservatives such as bacteriocins and H_2_O_2_ can be used in mayonnaise (Fialová et al., 2008).

The following examples clarify the application of some natural preservatives in mayonnaise: Adeli Milani, Mizani, Ghavami, and Eshratabadi (2014) considered the effectiveness of yellow mustard as a natural preservative. Mayonnaise samples formulated with 1% mustard paste showed an increased shelf life with a reduced microbial population. This result was attributed to the presence of 4‐hydroxybenzyl isothiocyanate known as sinalbin (a kind of aromatic glucosinolate substance) in yellow mustard.

Annatto is another preservative which holds antioxidant and antimicrobial properties and is used in mayonnaise. Annatto is basically used as a coloring agent in foodstuffs. The antimicrobial activity of annatto dye is due to several mono and sesquiterpenes. Yolmeh et al. (2014) found that the annatto dye was capable to lessen the population of *E. coli*, particularly when the temperature is 25°C. In this regard, the viability of *E. coli* reached zero during 12 days after inoculation at 25°C.

Rafiee, Barzegar, Sahari, and Maherani (2018) studied the effect of nanoliposomes (NLs) containing the phenolic compounds of pistachio green hull on the mayonnaise microbial and chemical quality. The results demonstrated that the samples containing NLs with 1,000 mg/kg of phenolic compounds had the lower thiobarbituric acid and peroxide values compared to the control samples and the samples having free phenolic compounds. In addition, NLs containing 1,000 mg/kg of phenolic compounds showed the highest inhibitory impact on fungal and total viable counts. Moreover, free phenolic compounds and NLs had a similar impact on lactic acid and *Enterobacteriaceae* bacteria. Other examples regarding the use of natural preservatives in mayonnaise are given in Table 2.

**Table 2:** The function of natural preservatives used in mayonnaise

Preservative	Concentration	Microorganism type	Storage	Initial count	Post‐treatment count	Total result	References
Chitosan glutamate	3 g/L	*Lactobacillus fructivorans*	5 and 25°C, 8 days	5–6 Log CFU/g	0 Log CFU/g	Chitosan was unsuccessful as a preservative at 25°C. It may be valuable as a preservative when combining with acetic acid and storage	Roller and Covill (2000)
Oregano essential oil	0.2% (v/v)	*Salmonella Enteritidis*	8 and 30 ℃, 24 hr	4 Log CFU/mL	>0.5 Log reduction until 4 hr at 30 ℃ and until 24 hr at 8 ℃	The use of oregano essential oil was able to decrease the *Salmonella Enteritidis* growth, an enteropathogen related to the foodborne disease egg byproducts	Da Silva and Franco (2012)
Baicalin	500 µg/g	Total plate count	10 and 20°C, 14 days	2.1 Log CFU/g	1.5 Log CFU/g	The antibacterial activity of baicalin was depended on the incubation temperature. The shelf life of mayonnaise kept at 10°C was meaningfully prolonged compared to products preserved at 20°C	Szymon et al. (2006)
Chitosan	100, 500 and 1,000 ppm	*Lactobacillus plantarum, Lactobacillus fructivorans, Serratia liquefaciens* or *Zygosaccharomyces bailli*	25°C	7 Log CFU/g	*L. plantarum*: short time inhibition *L. fructivorans*: rapidly killed *S. liquefaciens*: short time inhibition *Z. bailli*: completely growth inhibition	Antimicrobial effects of chitosan were depended on its molecular weight	Oh, Kim, Chang, and Kim (2001)
Gamma irradiation	1.5 kGy	Total count and total *coliforms*	2 days of irradiation, 37°C, 48 hr	Total count: 212.33 ± 194.06 CFU/g Total *coliform*: 50 ± 25.98 MPN/g	Not detected	The radiation dose required to decrease the *Salmonella* load as much as one log in eggs was 448 Gy suggested eggs irradiated with 1.5 kGy were microbiologically safe to prepare mayonnaise	Al‐Bachir and Zeinou (2006)

## 4 CONCLUSION

This review highlighted the factors affecting mayonnaise stability and texture which are under the effect of manipulation of mayonnaise formulation. Moreover, many ideas to produce healthy and functional mayonnaise sauce were discussed here. Every ingredient has a specific role, and the increase or decrease of any particle will affect the texture, stability, and sensory attributes, as well as antioxidant stability of the product. Taking this fact into account, many researchers successfully reduced oil content even up to 60% by replacement of oil for various stabilizers and this reduction of oil content of the product could decrease oxidation indirectly. This review had a quick look to the attempts made during two recent decades about reducing fat, using probiotics, prebiotics, and natural preservatives; decreasing cholesterol content; and adding nutritious supplements like phytosterol to prevent cholesterol adsorption. Overall, it could be noted that it is possible to design a nutritious and healthy mayonnaise with lower fat and cholesterol.

## CONFLICT OF INTERESTS

The authors declare that they have no conflict of interest.

## ETHICAL STATEMENT

The authors state that human and vertebrate animal testing was unnecessary in this study.
